# Prognostic factors for recurrence in acquired hemophilia A-results from a long-term observational study

**DOI:** 10.1016/j.rpth.2025.102707

**Published:** 2025-02-27

**Authors:** Lisa Reich, Florian Gatzke, Steffen Rauchfuss, Stefanie Roth, Wolfgang Miesbach

**Affiliations:** Medical Clinic 2, University Hospital Frankfurt, Germany

**Keywords:** acquired hemophilia, bleeding, factor VIII, recurrence, remission

## Abstract

**Objectives:**

Acquired hemophilia A (AHA) is a rare autoimmune disease caused by autoantibodies against factor (F)VIII (FVIII), potentially leading to life-threatening bleeding. While predictors for remission have been analyzed, data on recurrence is lacking.

**Methods:**

This study investigated predictors of AHA recurrence in 41 patients. Patients were divided into 2 groups: those with recurrence (*n* = 18) and those in stable long-term remission (*n* = 23) with at least 1 year of follow-up.

**Results:**

All relapses occurred within 1 year of initial remission. The median follow-up period was 3.8 years (IQR, 1.8-6.4) for all included patients. Multivariate Cox regression analysis revealed that initial FVIII activity <1 IU/dL and failure to achieve initial complete remission (CR) were significant predictors of relapse. Kaplan–Meier curves showed significantly different relapse-free survival rates for patients with initial FVIII activity <1 IU/dL vs ≥1 IU/dL (χ^2^[1] = 5.950, *P* = .015), and for those achieving initial CR vs partial remission (χ^2^[1] = 6.570, *P* = .010).

Other factors such as inhibitor titer, gender, age, World Health Organization scale, underlying disorder, controlled disorder, initial immunosuppressive therapy, immunosuppressive therapy escalation, and partial remission at day 21 showed no significant relation to recurrences. Overall survival did not differ significantly between relapsing and nonrelapsing patients (χ^2^[1] = .896, *P* = .344).

**Conclusion:**

Initial FVIII <1 IU/dL and failure to achieve initial CR are identified as risk factors for recurrence in AHA. Patients with these characteristics should be closely monitored for at least 1 year after initial remission due to increased recurrence risk.

## Essentials

• AHA: rare autoimmune disease with anti-FVIII antibodies.
• Study investigated predictors of AHA recurrence.
• Better relapse-free survival with initial FVIII ≥1 IU/dL and after achieving complete remission.
• Initial FVIII <1 IU/dL and failure to achieve complete remission predicted AHA relapses.

## 1 Introduction

Acquired hemophilia A (AHA) is a rare autoimmune disease (incidence of 1.48/million/year [1]), which is caused by autoantibodies against factor (F)VIII (FVIII) and can lead to potential life-threatening bleeding events. Diagnosis of AHA is not commonplace and should best be made by a hemostasis specialist [[2], [3], [4]]. AHA patients usually show subcutaneous and muscular hemorrhages clinically. Joint hemorrhages as typical of congenital hemophilia A [5], are rarely seen. Primary therapy goals are prevention and control of bleeding, inhibitor eradication by immunosuppression and therapy of a potential underlying disease [1,2,[6], [7], [8]]. AHA predominantly affects older patients with many comorbidities. Some patients have malignant or other autoimmune diseases (eg, rheumatoid arthritis, systemic lupus erythematosus). In rare cases, the disease occurs in connection with pregnancy. Most cases of AHA are still considered idiopathic [1,6,9,10].

The main immunosuppressive drugs currently used in AHA are steroids, cyclophosphamide (CTX) and rituximab (RTX). They are administered either alone or as a combination therapy [3,11,12]. Also worth mentioning is a relatively new combined therapy regimen called CyDRi (cyclophophymide, dexamethasone, and rituximab combined), which may help especially older, multimorbid AHA patients to achieve high remission rates and to suffer less adverse events caused by the toxicity of treatment [13]. Modern therapy recommendations suggest a combination of hemostatic and immunosuppressive treatment [2]. In the future, new treatment options with bispecific antibodies such as emicizumab will also play a role and my lead to a turning point in therapy [[14], [15], [16], [17], [18], [19]].

The primary goal of therapy is to achieve partial (PR) or better complete remission (CR) without subsequent recurrence. Partial remission is defined as no active bleeding after stopping any hemostatic drug for >24 hours and FVIII activity >50 IU/dL, while CR is achieved by patients in PR with negative inhibitor test, prednisolone tapered to <15 mg/day, and any other immunosuppressive therapy (IST) stopped [9]. CR rates range between 60% and 70% (GTH-AH 61% [9]; SACHA 61% [20]; UK surveillace study 71% [1]; EACH2 72% [6]).

While predictors for the achievement of remission have been investigated [9], recurrence data are lacking and only a few more or less clinically useful predictors could be found yet [[21], [22], [23], [24]]. Previous studies described that 20% to 30% of patients suffer from recurrences [1,9,20,25]. Due to the fact that recurrences can be potentially fatal or at least have an impact on the patient’s quality of life, efforts should be made to identify high-risk patients in advance to monitor them more closely. The aim in the future should be, to avoid recurrences, or at least to recognize them immediately such that appropriate treatment can be initiated as early as possible. Also many studies have the problem of a too short follow-up period after the initial AHA episode to even register all occurred recurrences.

Therefore, there is a need for long-term observational studies that closely examine baseline characteristics of relapsing patients and patients in long-term remission with focus on predictors of recurrences. Knowledge of recurrence predictors could improve monitoring during the follow-up period. Especially patients at risk of recurrence could benefit from known predictors.

## 2 Methods

The study was a long-term observational retrospective and in parts prospective study of 79 AHA patients treated between October 1995 and January 2021 at the Hemophilia Comprehensive Care Center of University Hospital Frankfurt am Main. The research protocol was approved by the ethics committee (approval number 20-1066).

### 2.1 Study population

AHA was defined by the presence of a FVIII activity <50 IU/dL and a neutralizing FVIII inhibitor ≥0.4 Bethesda units (BU)/mL. Definition of initial remission state was used according to the German, Austrian, and Swiss Thrombosis and Hemostasis Society (GTH) criteria. Recurrence was defined as renewed FVIII activity decrease to <50 IU/dL and renewed neutralizing FVIII inhibitor ≥0.4 BU/mL after initial CR or PR.

Patients under 18 years of age, patients with unknown or no remission (*n* = 22), and patients who died shortly after initial AHA diagnosis (*n* = 11) were excluded from further evaluation. Due to the fact that all 18 observed recurrences occurred within 1 year after initial PR or CR, patients with an observational period <1 year after initial PR or CR and no relapse also were excluded (*n* = 5). In total, 41 patients met the inclusion criteria and were included in further data analysis.

### 2.2 Data collection

Data collection was based on existing patient files at the hemophilia center Frankfurt a.M. and additional structured patient interviews. Patients who no longer had regular appointments at the hemophilia outpatient clinic were contacted about actual morbidity status and a supplementary structured patient interview was conducted with 13 patients after informed consent was obtained. All patients who no longer had regular follow-up appointments were contacted to verify their survival status. All patients without relapse were followed up by regular laboratory checks for FVIII and FVIII inhibitor at least 1 year after initial PR/CR. Those who could not be contacted and were followed up for <1 year were excluded from further analysis.

The following demographic and baseline characteristics of the patients were collected: date of and age at first diagnosis, gender, World Health Organization (WHO) scale, underlying disorder, comorbidities, observational period, initial FVIII:C, initial inhibitor concentration, initial hemoglobin value. Therapy data on the selected immunosuppressive treatment (dose, calendar dates of start and stop, need for therapy escalation), as well as outcome data (initial remission state [calendar date], relapse [calendar date, time from initial remission to relapse in days], death [calendar date]) were collected.

### 2.3 Statistical analysis

Statistical analyses were performed using IBM SPSS Statistics Version 29.0 (SPSS Inc, IBM Corp). To describe patients’ baseline characteristic in numbers and frequencies, descriptive statistics like medians and IQRs were used. To dichotomize the patient groups, dividers of previous studies (Tiede et al. [9]) were used.

The Fisher’s exact test was applied to compare categorical variables and the Wilcoxon–Mann–Whitney test was used for continuous variables. To evaluate the impact of variables, hazards ratios (HRs) with 95% CIs were calculated.

Univariate Cox regression analysis was performed for the occurrence of recurrence. The following predefined categorical and dichotomized variables were entered as independent variables in the univariate analysis: initial FVIII activity (<1 IU/dL vs ≥1 IU/dL), inhibitor concentration (≤20 BU/mL vs >20 BU/mL), gender, age at AHA first diagnosis (≤74 years vs > 74 years), WHO scale (good [≤2] vs poor [>2]), controlled underlying disorder (yes vs no), initial IST (steroids only vs steroids plus), initial therapy escalation of IST (yes vs no), initial remission at day 21 after initial diagnosis (no remission vs PR) initial remission state (PR vs CR). Subsequently, a multivariate analysis for the occurrence of recurrence was carried out with initial FVIII activity (<1 IU/dL vs ≥1 IU/dL) and initial remission state (PR vs CR). The same procedure was carried out for overall survival.

To estimate relapse-free survival rates and differences in overall survival of patients with/without recurrence Kaplan–Meier analysis was used. Differences between curves were assessed using the log-rank test.

A *P* value < .05 was considered statistically significant for all performed analyses.

## 3 Results

### 3.1 Baseline characteristics

Baseline characteristics and demographics of the included 41 patients are summarized in Table 1. The median age at first diagnosis was 71 years (IQR, 57-77) and gender distribution was nearly balanced. Most patients had good WHO scale ≤2 (78%). Median follow-up period was 3.8 years (IQR, 1.8-6.4) for all included patients. Further information is provided in Table 1.

**Table 1:** Baseline characteristics.

Baseline characteristics	All patients (n=41)
Age at first diagnosis in years, median (IQR)	71 (57-77)
Gender	
Male, *n* (%)	22 (54)
Female, *n* (%)	19 (46)
WHO scale, *n* (%)	
0	20 (49)
1	6 (15)
2	6 (15)
3	4 (10)
4	5 (12)
Underlying disorder, *n* (%)	
None/idiopathic	27 (66)
Malignancy	6 (15)
Autoimmunity	3 (7)
Postpartum	5 (12)
Comorbidities, *n* (%)	
Cardiovascular (Heart failure, CAD, PAOD)	15 (37)
Renal failure	8 (20)
Liver failure	7 (17)
Arterial hypertension	27 (66)
Diabetes mellitus type 2	9 (22)
Neurologic disorder (Alzheimer’s disease, Parkinson’s disease, hemiparesis after stroke)	4 (10)
Observation period in years, median (IQR)	3.8 (1.8-6.4)
Initial FVIII:C in IU/dL, median (IQR)	2.6 (0.9-7.2)
Initial FVIII:C < 1 IU/mL, *n* (%)	11 (28)
Initial inhibitor concentration in BU/mL, median (IQR)	11.8 (3.6-33.6)
Initial inhibitor concentration > 20 BU/mL, *n* (%)	15 (38)
Initial hemoglobin minimum in g/dL, median (IQR)	8.9 (7.7-12.1)

CAD, coronary artery disease; PAOD, peripheral arterial occlusive disease; WHO, World Health Organization.

### 3.2 Bleeding, hemostatic treatment, and hospitalization in recurrence

During the first recurrence, 7 out of 18 patients (38.9%) experienced bleeding. In the overall cohort (*n* = 79), bleeding was observed in 96% (*n* = 76) during the initial AHA episode. For nearly all patients, bleeding symptoms prompted the initial AHA diagnosis, with only 3 patients (3.8%) being diagnosed due to coagulation abnormalities detected prior to surgery. Among the remaining 11 patients who did not experience bleeding during relapse, recurrence was detected solely through regular laboratory tests conducted during the follow-up period.

In recurrence, 5 of the hemorrhaging patients required a transfusion of at least 1 erythrocyte concentrate, of which 2 received additional bypass therapy. Susoctocog alfa therapy (OBIZUR) was given to 2 bleeding patients in recurrence.

Among the bleeding patients, 4 exhibited multiple hematomas on the extremities, one experienced macrohematuria, another had recurrent shunt hemorrhages (classified as muscular hemorrhage and subcutaneous hematoma), and 1 patient presented with multiple hematomas combined with gastrointestinal bleeding and hemorrhage following bone marrow aspiration (Table 2).

**Table 2:** Initial bleeding pattern versus bleeding pattern at recurrence.^a^

Bleeding symptoms	Initial bleeding *n* = 76	Bleeding 1st relapse *n* = 7
Hematoma, *n* (%)	55 (72.4)	6 (85.7)
Muscular bleeding, *n* (%)	37 (48.7)	1 (14.3)
Urogenital bleeding, *n* (%)	12 (15.8)	1 (14.3)
Gastrointestinal bleeding, *n* (%)	8 (10.5)	1 (14.3)

a It should be noted that some patients had several bleeding manifestations.

The nonbleeding relapsing patients were managed as outpatients, whereas those with hemorrhage required hospitalization. Comparatively, during the initial AHA episode, 72 out of 79 patients (91.1%) needed hospitalization.

### 3.3 Outcome

**Figure 1:**
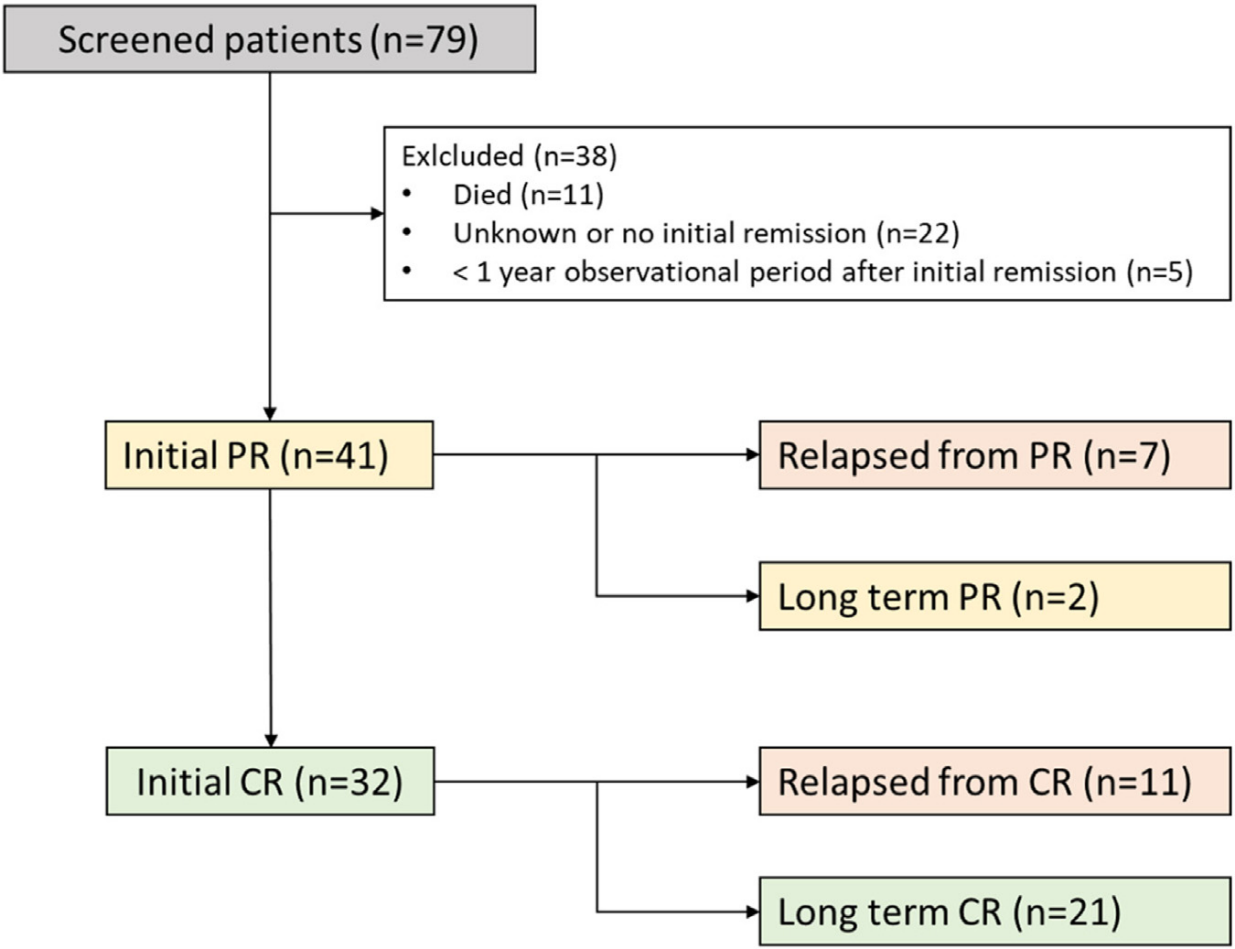
Initial remission process. CR, complete remission; PR, partial remission.

Figure 1 shows the initial remission process of the analyzed patients. After primary AHA episode 32 (78%) patients achieved initial CR, while 9 (22%) remained in initial PR. Initial CR was achieved after a median of 69 days (IQR, 48-105) and initial PR after 28 days (IQR, 16-55). Time from first diagnosis to initial PR (*P* = .581) and CR (*P* = .650) did not differ between patients with or without recurrence.

In total, 7 patients recovered from initial PR and 11 from initial CR. All relapsing patients had their first relapse <1 year after achieving initial remission (PR or CR). The primary endpoint of recurrence was reached by 18 (44%) patients. Median time in remission was 49 days after initial remission. More precise information to time in remission is provided in Table 3. One year after initial remission, 2 patients remained in long-term PR and 21 patients in long-term CR.

**Table 3:** Relapse after initial remission as primary endpoint and overall survival as secondary endpoint of the study.

Endpoint	Endpoint achieved, *n* (%)	Time to endpoint in days (mo; y)		
		Median	IQR	Range
**Primary endpoint**				
Relapse after initial remission	18 (44)	49 (2; 0)	22-107 (1-4; 0-0)	8-353 (0-12; 0-1)
**Secondary end point**				
Mortality	11 (28)^a^	1164 (56; 3)	426-1691 (15-56; 1-5)	198-3716 (7-122; 1-10)

a The survival state of 2 patients was unknown at final follow-up. With regards to the overall survival analysis, these 2 patients were excluded. Percentages therefore refer to a total of 39 patients (100.0%).

At final follow-up, 28 (72%) of the patients were alive. The 1-year mortality of the included patients was 4.9% (*n* = 2). While interpreting the low 1-year mortality rate of this study, it is important to note that patients who died immediately after initial AHA diagnosis (*n* = 11) or could not be followed up for 1 year for other reasons (*n* = 5) were excluded from further analysis due to the aim of investigating predictors of recurrence (long-term remission vs recurrence). All patients without relapse were followed up by regular laboratory checks for FVIII and FVIII inhibitor (objectified relapse parameters) at least 1 year after initial PR/CR (median, 1.8 years).

### 3.4 Predictors of recurrence

Recurrence according to baseline characteristics is shown in Table 4. Statistical significance of univariate Cox regression analysis was achieved for FVIII activity <1 IU/dL and initial CR. Patients with initial FVIII activity <1 IU/dL and no initial CR (PR; ongoing remission) are more likely to relapse. Both factors were entered into multivariate Cox regression analysis and remained significant (Table 5). Inhibitor titer, gender, age, WHO scale, underlying disorder, controlled disorder, initial IST, IST escalation, and PR at day 21 showed no significant relation to recurrences. Due to small sample sizes per IST treatment group (prednisolone only, *n* = 24; prednisolone plus cyclophosphamide, *n* = 19; prednisolone plus cyclophosphamide plus rituximab, *n* = 2; rituximab only, *n* = 1) the patients were summarized in the prednisolone-only group and the prednisolone plus cyclophosphamide ± RTX group. Also the need for therapy escalation during the initial AHA episode due to insufficient initial IST was analyzed. For both variables, no significant relation to the occurrence of recurrence was found.

**Table 4:** Relapse corresponding to baseline characteristics: univariate analysis.

Baseline variable	Relapse suffered, *n* (%)	Remission to R1 in days, median (IQR)	Unadjusted HR (CI)
Factor VIII activity			
< 1 IU/dL, *n* = 11	8 (73)	60 (20-140)	0.32 (0.12-0.84)^a^
≥ 1 IU/dL, *n* = 29	9 (31)	49 (25-95)	
Inhibitor concentration			
≤ 20 BU/mL, *n* = 24	9 (38)	40 (22-87)	1.48 (0.57-3.84)
> 20 BU/mL, *n* = 15	8 (53)	77 (22-140)	
Gender			
Female, *n* = 19	5 (26)	55 (25-95)	2.67 (0.94-7.58)
Male, *n* = 22	13 (59)	45 (20-136)	
Age at AHA diagnosis			
≤ 74 y, *n* = 29	11 (38)	55 (25-127)	2.01 (0.76-5.31)
> 74 y, *n* = 12	7 (58)	49 (21-83)	
WHO scale			
Good (≤ 2), *n* = 32	13 (41)	55 (21-107)	1.30 (0.42-4.00)
Poor (> 2), *n* = 9	5 (56)	43 (26-129)	
Underlying disorder			
Autoimmunity, *n* = 3	1 (33)	-	
Malignancy, *n* = 6	3 (50)	-	
Postpartum, *n* = 5	1 (20)	-	
None/idiopathic, *n* = 27	13 (48)	52 (23-113)	
Underlying disorder controlled^b^			
Yes, *n* = 3	2 (67)	-	2.34 (0.33-16.73)
No, *n* = 6	2 (33)	-	
Initial IST			
Prednisolone, *n* = 24	11 (46)	63 (22-127)	1.05 (0.36-3.09)
Prednisolone +, *n* = 12	5 (42)	36 (20-62)	
Initial therapy escalation			
Yes, *n* = 13	5 (38)	36 (16-62)	0.89 (0.31-2.53)
No, *n* = 27	13 (48)	63 (23-142)	
Initial remission at day 21 after initial diagnosis			
No remission, *n* = 23	12 (52)	55 (20-136)	0.57 (0.20-1.62)
Initial PR, *n* = 15	5 (33)	49 (29-87)	
Initial CR			
Yes, *n* = 32	11 (34)	45 (26-92)	0.30 (0.11-0.80)^a^
No (= PR), *n* = 9	7 (78)	70 (20-150)	

CR, complete remission; IST, immunosuppressive therapy; PR, partial remission; WHO, World Health Organization.

a Statistical significance of univariate Cox regression analysis with *P* < .05.

b Controlled means no progress in case of malignancy (*n* = 1) or no flare up in case of autoimmunity in course of follow-up (*n* = 2); 2 patients in this group suffered a relapse, postpartum and idiopathic were excluded, because they are automatically “controlled.”

**Table 5:** Predictors of recurrence: multivariate analysis.

Baseline variable	Relapse, HR (CI)
Factor VIII activity <1 IU/dL	0.37 (0.14-0.98)
Initial complete remission	0.35 (0.13-0.93)

HR, hazard ratio.

### 3.5 Relapse-free survival

**Figure 2:**
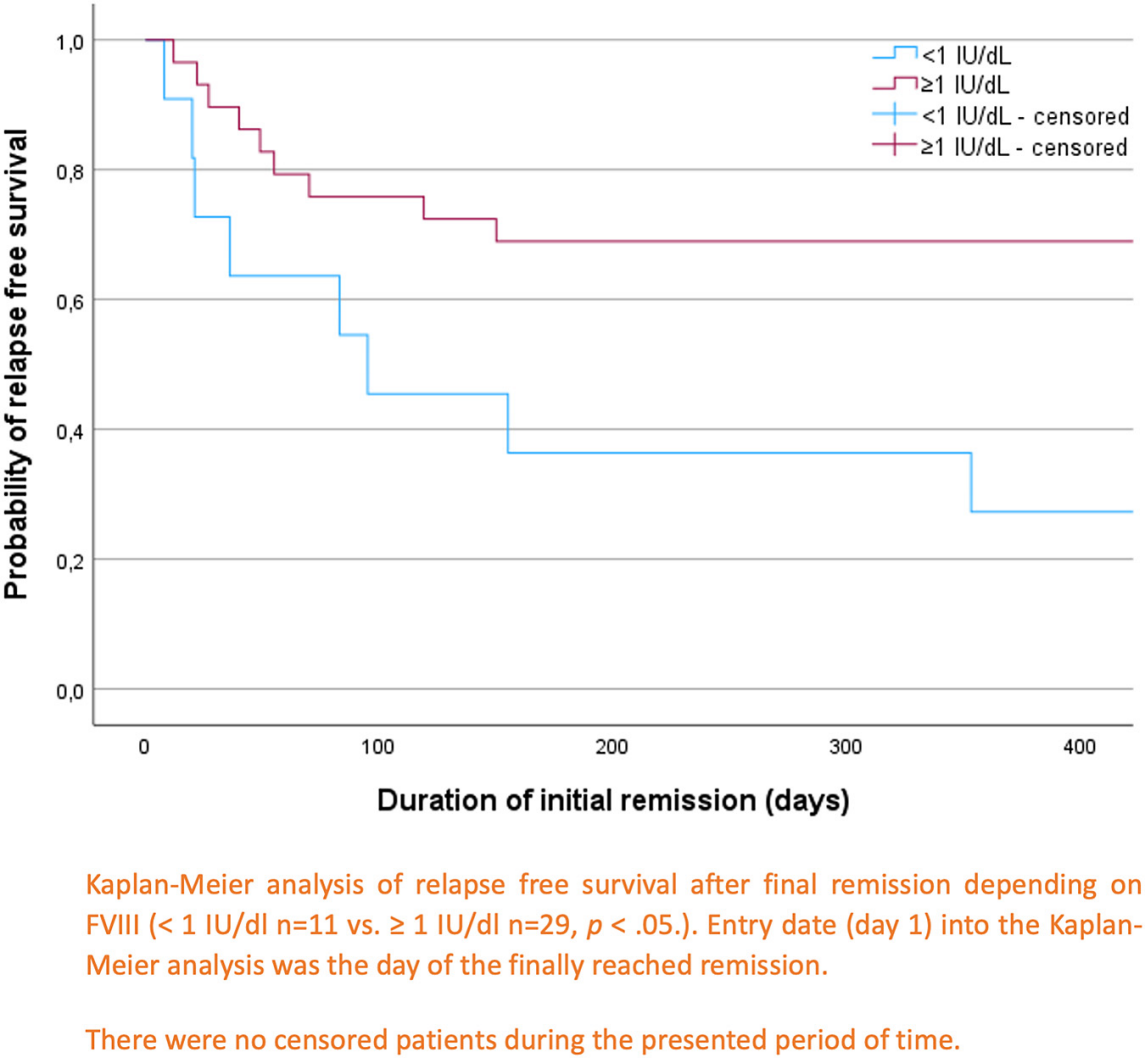
Relapse-free survival depending on factor (F)VIII activity <1 IU/dL versus FVIII activity ≥1 IU/dL.

**Figure 3:**
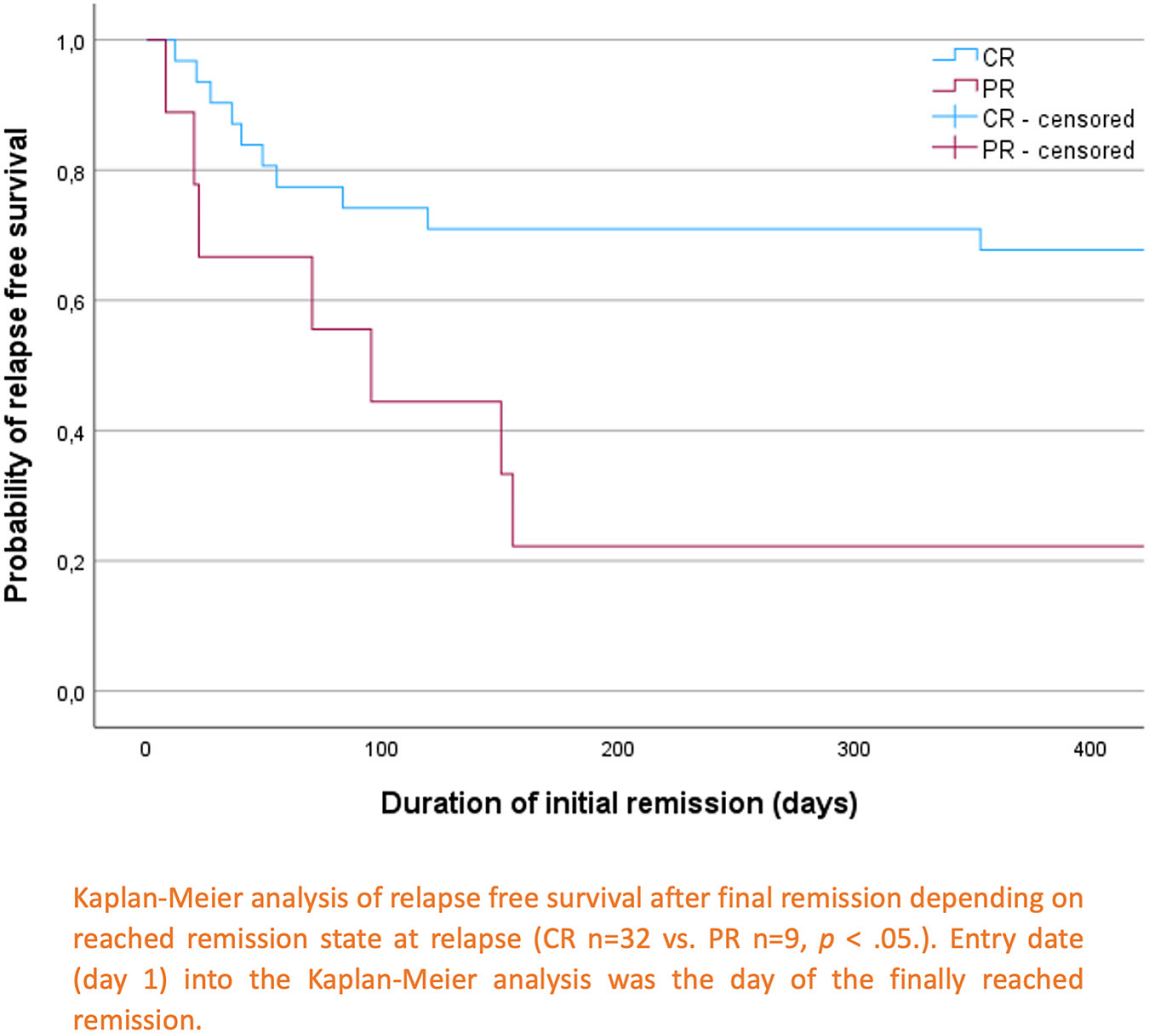
Relapse-free survival depending on initial remission state (partial remission vs complete remission).

To estimate relapse-free survival rates according to initial FVIII activity <1 IU/dL and achievement of initial CR, Kaplan–Meier analyses were used and log-rank tests were performed to assess differences. The date of entry into the Kaplan–Meier analyses (shown in Figures 2 and 3) was the date of the finally reached remission. Curves of relapse-free survival of patients with initial FVIII activity <1 IU/dL and patients with initial FVIII activity ≥1 IU/dL differed significantly (Figure 2), χ^2^(1) = 5.950, *P* = .015. The same applied to relapse-free survival of patients with initial CR versus patients with initial PR/ongoing remission (Figure 3), χ^2^(1) = 6.570, *P* = .010.

Also the effect of initial immunosuppressive treatment (“prednisolone only” vs “prednisolone plus cyclophosphamide”) on remission time (PR/CR) was analyzed. The results indicated no significant differences between the 2 groups, *P* = .244 (PR to R1) and *P* = .327 (CR to R1).

Overall, no significant differences were found in the times to achieve a PR or CR during the initial AHA episode compared to the times to achieve a second PR/CR in the relapse, *P* = .295 (PR) and *P* = .193 (CR).

### 3.6 Recurrence and overall survival

As shown above for recurrence (Table 4) univariate Cox regression was performed for overall survival. WHO scale >2 and underlying malignant disease were statistical significantly associated with poor overall survival (not shown). Both factors were entered in multivariate regression and remained significant. Initial FVIII activity, inhibitor titer, gender, age, controlled disorder, initial IST, IST escalation, and initial CR and PR at day 21 showed no significant relation to overall survival. Again, it is important to note that patients who died immediately after initial AHA diagnosis (*n* = 11) were excluded from the study. Overall mortality rate for the complete observational time (database closure, 01/2021) of the cohort was 28% (*n* = 11), 35% (*n* = 6) for the relapsing patients, and 23% (*n* = 5) for the nonrelapsing subgroup, *P* = .482.

General 1-year mortality was 5% (*n* = 2), 11% (*n* = 2) for relapsing patients, and 0% (*n* = 0) for nonrelapsing patients, *P* = .187. The survival state of 2 patients was unknown at final follow-up. With regards to the overall survival analysis, these 2 patients were excluded. At 1-year follow-up, both patients were alive and therefore included in the analysis of 1-year mortality.

**Figure 4:**
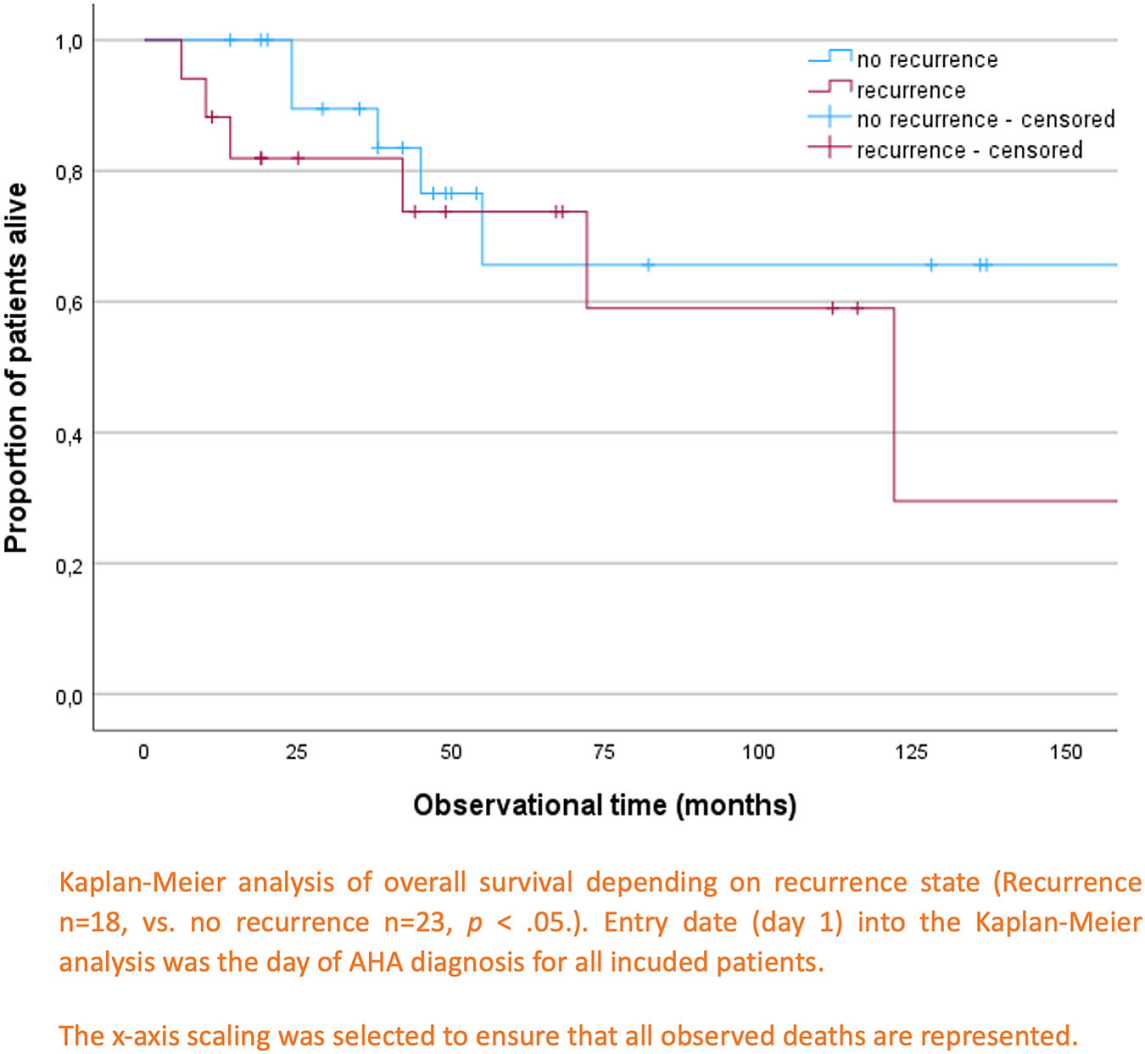
Overall survival of patients with versus without recurrence.

To estimate overall survival of patients in long-term stable remission vs patients with at least 1 relapsing episode, additional Kaplan–Meier analysis was used and log-rank test was performed to assess a difference. The entry date for the overall survival analysis is the date of initial AHA diagnosis for all patients. The curves for overall survival of patients with versus without recurrence of AHA showed no significant difference (Figure 4), χ^2^(1) = 0.918, *P* = .338. The x-axis scaling in Figure 4 was selected to depict all observed deaths.

## 4 Discussion

This study is the first to investigate possible predictors of recurrence in AHA. So far, there is no study that identified clinically useful predictors of recurrences in AHA and can demonstrate a comparably long observational period with a median of 3.8 years. We showed that initial FVIII activity <1 IU/dL is a possible predictor of recurrence, while the achievement of initial CR proved to be a protective factor against recurrences.

While only a few practical useful predictors for recurrence have been analyzed, prognostic parameters for achieving remission and overall survival have been identified by Tiede et al. [9]. In that study, baseline FVIII activity <1 IU/dL was associated with poor survival and longer time to achieve PR or CR [9]. These results are consistent with our findings and speak for the validity of our data. Our results also support the model of defining subgroups of patients with better prognosis and outcome, depending on initial FVIII activity and the achievement of initial CR.

Only a few previous studied had investigated predictors of recurrence and the effect of the appearance of AHA recurrence on overall survival [24]. For example, circulating anti-IgA autoantibodies could be found as potential predictors of poor recurrence-free survival and poor overall survival in AHA patients [23]. Due to the retrospective study design, it was not possible to analyze the antibody subgroups for this study population. A potential risk elevation in patients with autoimmune [26] or lymphoproliferative disorders [24] or pregnancy-related AHA [22] could not be investigated due to the limited number of cases. Another study also suggests monitoring the FVIII:C/VWF:Ag ratio as tool of relapse prediction [21].

The fact that overall survival of patients with and without relapses showed no significant difference, can speak in favor of the applied aftercare concept. The vast majority of patients in this study were closely monitored clinically and by coagulation analysis after the following concept: weekly appointments for the first month after discharge from inpatient treatment, then fortnightly appointments for the second month, then monthly appointments till 6 months and 2 to 3 monthly appointments till 12 months. After 1 year, 6-monthly to annual check-ups were carried out. If complications or a more complicated course of the disease occurred, more frequent check-ups were arranged. This procedure largely corresponds to the follow-up concept recommended internationally in 2020 [9,27]. The low hospitalization rate compared to the initial episode and the fact that many patients only experienced subclinical recurrences and were not again exposed to serious bleeding manifestations also speaks in favor of the applied follow-up concept. Maybe a combination of the results of this and the mentioned studies above [[21], [22], [23], [24],^26^] could prevent relapses in future patients. For example, patients at high risk (especially patients with initial FVIII <1%; patients with a lymphoproliferative disorder) could be selected for closer monitoring by using FVIII:C/VWF:Ag ratio and by analyzing the immunoglobulin G subclasses of FVIII autoantibodies as supplementary laboratory markers. This would make sense, because FVIII:C is routinely determinated at first diagnosis of AHA and FVIII:C/VWF:Ag ratio and the antibody subgroup analysis are not. As already indicated above, step-by-step diagnostics would be conceivable for the future.

Nevertheless, this study also has its limitations. First of all, the study is monocentric and in most parts retrospective. The monocentric character also can be seen as advantage because of the continuity of therapy due to a homogeneous treatment and follow-up concept over the period of the study by the treating physicians. Because of the observational character recall bias, selection bias, confounding, and inconsistencies in data collection cannot be excluded.

To test the validity of our data, we compared the baseline characteristics of our collective with 2 large AHA studies—the EACH2-registry (2003-2008) and the GTH-AH study (2010-2013). Overall, most patients’ characteristics like gender, initial FVIII activity, initial inhibitor titer, and initial hemoglobin level are comparable with both of these studies. In comparison, the following differences emerged: patients of this study were younger (median 71 years vs 74 years [EACH2]), had less serious vascular diseases and showed a lower WHO scales. These differences and the low 1-year mortality and overall mortality rate can be explained by the exclusion of patients who died during the initial AHA episode, never achieved remission, or weren’t followed up for at least 1 year after initial remission. Inclusion of these patients would make little sense in terms of an analysis based on a longer-term remission assessment, but makes a selection bias possible. It should also be mentioned that most of the patients analyzed were included between 2008 and 2018, which may also explain the low mortality rate despite the high entry age of some of the patients. Also more patients with idiopathic and postpartum AHA were included in this study. Another major difference and advantage at the same time is the longer objectivated observational period of a median of 1.8 years (EACH2: 1.0 years [6]; GTH-AH: 0.7 years [9]).

The evolving landscape of immunosuppression strategies in AHA has significantly impacted treatment approaches since this study was conducted. The GTH study has conclusively demonstrated the high toxicity and suboptimal CR rates associated with the traditional steroids plus cyclophosphamide regimens [9]. As a result, there has been a paradigm shift toward rituximab-based immunosuppression, which has gained favor among many centers due to its lower toxicity profile [13,28]. While rituximab may lead to longer remission times, the introduction of emicizumab for bleeding prophylaxis has effectively addressed this concern, allowing for more flexible treatment strategies [18,19]. These advancements underscore that while the study’s findings remain informative, they may not accurately represent current clinical practices. Future research should focus on evaluating the long-term outcomes of these newer treatment approaches to provide a more up-to-date and comprehensive understanding of AHA management.

In conclusion, this study shows clinically useful predictors of recurrences in AHA. In addition, thanks to the long follow-up period; it was possible to determine that all observed recurrences occurred within the first year after initial remission. This once again highlights the importance of a continuous follow-up of at least 1 year after initial remission. With regards to the results of the study, particular attention should be paid to patients with an initial FVIII activity <1 IU/dL and patients who did not achieve initial CR.

## References

[1] Collins P.W., Hirsch S., Baglin T.P., Dolan G., Hanley J., Makris M. (2007). Acquired hemophilia A in the United Kingdom: a 2-year national surveillance study by the United Kingdom Haemophilia Centre Doctors’ Organisation. Blood.

[2] Tiede A., Collins P., Knoebl P., Teitel J., Kessler C., Shima M. (2020). International recommendations on the diagnosis and treatment of acquired hemophilia A. Haematologica.

[3] Fragner M., Imbo B., Hobson J., Roberts J.C., Rajasekhar A., Tarantino M.D. (2022). Time is blood: the impact of diagnostic delays on acquired hemophilia A. Cureus.

[4] Tiede A., Giangrande P., Teitel J., Amano K., Benson G., Nemes L. (2019). Clinical evaluation of bleeds and response to haemostatic treatment in patients with acquired haemophilia: a global expert consensus statement. Haemophilia.

[5] Bolton-Maggs P.H.B., Pasi K.J. (2003). Haemophilias A and B. Lancet.

[6] Knoebl P., Marco P., Baudo F., Collins P., Huth-Kühne A., Nemes L. (2012). Demographic and clinical data in acquired hemophilia A: results from the European Acquired Haemophilia Registry (EACH2). J Thromb Haemost.

[7] Hay C.R.M., Brown S., Collins P.W., Keeling D.M., Liesner R. (2006). The diagnosis and management of factor VIII and IX inhibitors: a guideline from the United Kingdom Haemophilia Centre Doctors Organisation. Br J Haematol.

[8] Tiede A., Scharf R.E., Dobbelstein C., Werwitzke S. (2015). Management of acquired haemophilia A. Hamostaseologie.

[9] Tiede A., Klamroth R., Scharf R.E., Trappe R.U., Holstein K., Huth-Kühne A. (2015). Prognostic factors for remission of and survival in acquired hemophilia A (AHA): results from the GTH-AH 01/2010 study. Blood.

[10] Green D., Lechner K. (1981). A survey of 215 non-hemophilic patients with inhibitors to factor VIII. Thromb Haemost.

[11] Kruse-Jarres R., Kempton C.L., Baudo F., Collins P.W., Knoebl P., Leissinger C.A. (2017). Acquired hemophilia A: updated review of evidence and treatment guidance. Am J Hematol.

[12] Huth-Kühne A., Baudo F., Collins P., Ingerslev J., Kessler C.M., Lévesque H. (2009). International recommendations on the diagnosis and treatment of patients with acquired hemophilia A. Haematologica.

[13] Simon B., Ceglédi A., Dolgos J., Farkas P., Gaddh M., Hankó L. (2022). Combined immunosuppression for acquired hemophilia A: CyDRi is a highly effective low-toxicity regimen. Blood.

[14] Pfrepper C., Klamroth R., Oldenburg J., Holstein K., Eichler H., Hart C. (2024). Emicizumab for the treatment of acquired hemophilia A: consensus recommendations from the GTH-AHA working group. Hamostaseologie.

[15] Kitazawa T., Igawa T., Sampei Z., Muto A., Kojima T., Soeda T. (2012). A bispecific antibody to factors IXa and X restores factor VIII hemostatic activity in a hemophilia A model. Nat Med.

[16] Knoebl P., Thaler J., Jilma P., Quehenberger P., Gleixner K., Sperr W.R. (2021). Emicizumab for the treatment of acquired hemophilia A. Blood.

[17] Möhnle P., Pekrul I., Spannagl M., Sturm A., Singh D., Dechant C. (2019). Emicizumab in the treatment of acquired haemophilia: a case report. Transfus Med Hemother.

[18] Tiede A., Kemkes-Matthes B., Knöbl P. (2021). Should emicizumab be used in patients with acquired hemophilia A?. J Thromb Haemost.

[19] Tiede A., Hart C., Knöbl P., Greil R., Oldenburg J., Sachs U.J. (2023). Emicizumab prophylaxis in patients with acquired haemophilia A (GTH-AHA-EMI): an open-label, single-arm, multicentre, phase 2 study. Lancet Haematol.

[20] Borg J.Y., Guillet B., Le Cam-Duchez V., Goudemand J., Lévesque H., SACHA Study Group (2013). Outcome of acquired haemophilia in France: the prospective SACHA (Surveillance des Auto antiCorps au cours de l’hémophilie Acquise) registry. Haemophilia.

[21] Trossaert M., Graveleau J., Thiercelin-Legrand M.-F., Sigaud M., Guerrero F., Neel A. (2019). The factor VIII:C/VWF:ag ratio as a useful tool to predict relapse in patients with acquired haemophilia A: a retrospective cohort study. Haemophilia.

[22] Dewarrat N., Gavillet M., Angelillo-Scherrer A., Naveiras O., Grandoni F., Tsakiris D.A. (2021). Acquired haemophilia A in the postpartum and risk of relapse in subsequent pregnancies: a systematic literature review. Haemophilia.

[23] Tiede A., Hofbauer C.J., Werwitzke S., Knöbl P., Gottstein S., Scharf R.E. (2016). Anti-factor VIII IgA as a potential marker of poor prognosis in acquired hemophilia A: results from the GTH-AH 01/2010 study. Blood.

[24] Mizrahi T., Doyon K., Dubé E., Bonnefoy A., Warner M., Cloutier S. (2019). Relapse pattern and long-term outcomes in subjects with acquired haemophilia A. Haemophilia.

[25] Baudo F., Collins P., Huth-Kühne A., Lévesque H., Marco P., Nemes L. (2012). Management of bleeding in acquired hemophilia A: results from the European Acquired Haemophilia (EACH2) Registry. Blood.

[26] Salaj P., Geierová V., Ivanová E., Loužil J., Pohlreichová V., Hrachovinová I. (2020). Identifying risk factors and optimizing standard of care for patients with acquired haemophilia A: results from a Czech patient cohort. Haemophilia.

[27] Collins P., Baudo F., Knoebl P., Lévesque H., Nemes L., Pellegrini F. (2012). Immunosuppression for acquired hemophilia A: results from the European Acquired Haemophilia Registry (EACH2). Blood.

[28] Lévesque H., Viallard J.F., Houivet E., Bonnotte B., Voisin S., Le Cam-Duchez V. (2024). Cyclophosphamide vs rituximab for eradicating inhibitors in acquired hemophilia A: a randomized trial in 108 patients. Thromb Res.

